# A Comparative Study of Short-Term Vascular and Stromal Alterations of the Choroid Following Half-Fluence Photodynamic Therapy in Pachychoroid Neovasculopathy and Chronic Central Serous Chorioretinopathy

**DOI:** 10.3390/life12091304

**Published:** 2022-08-25

**Authors:** Özge Yanık, Sibel Demirel, Figen Batıoğlu, Emin Özmert

**Affiliations:** Department of Ophthalmology, Ankara University School of Medicine, 06620 Ankara, Turkey

**Keywords:** choroidal vascularity index, chronic central serous chorioretinopathy, half-fluence photodynamic therapy, optical coherence tomography, pachychoroid neovasculopathy

## Abstract

Background: Central serous chorioretinopathy (CSCR) and pachychoroid neovasculopathy (PNV) are among the pachychoroid spectrum diseases (PSDs). Half-fluence photodynamic therapy (hf-PDT) is one of the effective treatment methods for both diseases. The aim of this study was to compare the effect of hf-PDT on the choroidal structure in CSCR and PNV. Methods: This study included 35 patients with chronic CSCR and 18 patients with PNV. The hf-PDT protocol was applied to all eyes. Before and 3 months after hf-PDT, enhanced-depth optical coherence tomography images were analyzed. The total choroidal area (CA), luminal area (LA), and stromal area (SA) were measured using ImageJ software. Results: Compared with baseline values, 3 months after hf-PDT, the mean CA reduced from 1.398 to 1.197 mm^2^ (*p* < 0.001) in the CSCR group and the total CA reduced from 1.050 to 1.000 mm^2^ (*p* < 0.021) in the PNV group. The mean percentage changes in CA, LA, and SA values were statistically higher in the chronic CSCR group (13.86%, 13.53%, and 14.11%, respectively) than those in the PNV group (4.61%, 4.02%, and 5.74%; *p* = 0.001, *p* = 0.002, and *p* = 0.031, respectively). Conclusion: CSCR and PNV are thought to be PSDs. However, they differ in choroidal morphological response after hf-PDT, which might be a result of the different structural components of the PNV lesions.

## 1. Introduction

The concept of pachychoroid spectrum diseases (PSDs) was proposed within the past decade [1]. Central serous chorioretinopathy (CSCR) and pachychoroid neovasculopathy (PNV) are among the diseases in this spectrum [2]. Typical pachychoroid spectrum features include choroidal hyperpermeability, choroidal thickening, and dilated choroidal vessels (pachyvessels), with reduced overlying choriocapillaris [2]. In CSCR, there is a serous detachment of the neurosensory retina, generally associated with an underlying serous elevation of the retina pigment epithelium (RPE). In PNV, there is type 1 choroidal neovascularization (CNV) located under flat irregular pigment epithelial detachments (PEDs) with no evidence of age-related macular degeneration (AMD), myopic degeneration, or other degenerative processes [3]. However, the current literature includes different definitions for PNV [4]. A clear-cut definition to indicate the difference between PNV and CSCR complicated by CNV is lacking because the patient’s history or signs of a previous CSCR attack might not always be obvious. Overall, choroidal thickening or Haller vessel dilation and its effects on the inner choroidal layers seem to be an inductive factor in the pathogenesis of both diseases [2].

Photodynamic therapy (PDT) with verteporfin is an effective treatment method for chronic CSCR. Its main mechanism of action depends on its angio-occlusive properties, causing the constriction of choroidal vessels and choroidal vascular remodeling [5,6]. It reduces choroidal vascular hyperpermeability and exudation and induces the resorption of subretinal fluid. Half-fluence (hf) [7] and half-dose (hd) [8] PDT protocols have been proposed to avoid the potential side effects of a full-dose protocol, such as choriocapillaris ischemia, RPE atrophy, and CNV. In chronic CSCR cases, both half-fluence and half-dose PDT protocols have been used for a long time with proven effectiveness [9]. Hf-PDT reduces choroidal thickness by decreasing the diameter of large-caliber vessels and spares choriocapillaris flow [10]. However, current data regarding the use of PDT in the treatment of PNV are very limited [11,12,13,14]. A few studies have evaluated the treatment outcomes of PDT in PNV, and the authors reported that PDT may produce favorable results in the treatment of PNV [11,12,13,14].

Several previous studies of choroidal alterations after PDT evaluated subfoveal choroidal thickness (SFCT) as an indicator of treatment effectiveness on the choroid [15,16]. The recent development of sophisticated computer programs for image analysis has enabled two-dimensional choroidal analysis. In this type of analysis, two different compartments of the choroid, the lumen and stroma, are separately evaluated with binarization methods [17]. Furthermore, the choroidal vascularity index (CVI), a choroidal vascularity indicator based on binarized measurements, was recently developed and has several advantages over choroidal thickness measurements, such as high reliability and not being affected by age or ocular and systemic parameters [18,19].

To the best of our knowledge, no study has evaluated choroidal structural alterations after hf-PDT using the binarization method in PNV and comparing these choroidal changes with CSCR cases without CNV. The changes in the choroidal morphology in response to hf-PDT may vary in these two distinct groups, despite sharing common pachychoroid features. Therefore, the aim of this study was to evaluate the effect of hf-PDT on choroidal structure in chronic CSCR and PNV to develop a better understanding of the underlying pathophysiological mechanisms.

## 2. Materials and Methods

This retrospective comparative study included 18 treatment naïve eyes of 18 patients diagnosed with PNV and 35 treatment naïve eyes of 35 patients diagnosed with chronic CSCR without CNV who underwent hf-PDT. This study was approved by the Institutional Review Board Committee and conducted in accordance with the Declaration of Helsinki.

The diagnosis of PNV and chronic CSCR was confirmed with clinical examination and multimodal imaging modalities, including spectral domain optical coherence tomography (SD-OCT) (Spectralis, Heidelberg Engineering, Inc., Heidelberg, Germany), fundus fluorescein angiography (FA), indocyanine green angiography (ICGA), fundus autofluorescence (FAF) (Heidelberg Retina Angiograph 2, Heidelberg Engineering, Heidelberg, Germany), and optical coherence tomography angiography (OCTA) (AngioVue software of the RTVue XR Avanti, OptoVue, Inc., Fremont, CA, USA). Pachychoroid features were defined as the presence of choroidal hyperpermeability on ICGA, diffuse or focal increases in choroidal thickness, and large choroidal vessels compressing the choriocapillaris and Sattler’s layer. The inclusion criteria for chronic CSCR cases were the presence of foveal subretinal fluid on horizontal SD-OCT B-scan images showing persistence longer than six months and fluorescein leakage on FA, along with RPE window defects compatible with chronic CSCR with areas of corresponding hypercyanescence visible on ICGA. The presence of type 1 CNV and typical pachychoroid features in the absence of characteristic AMD or degenerative changes was defined as PNV. The diagnosis of PNV was given if the following criteria were met: flat irregular pigment epithelial detachment causing “double layer sign” on horizontal SD-OCT B-scan images, clearly visible neovascular tangled network on OCTA, and hypercyanescent plaque appearance on late-phase ICGA images.

Patients with any chorioretinal and/or inflammatory ocular diseases, patients with media opacities preventing adequate imaging, patients having a refractive error ≥ 5.00 diopter spherical equivalent, patients with low quality SD-OCT images below the manufacturer recommended signal index (15 Q) and/or low quality OCTA images below 6/10 scan quality, patients with a history of previous therapy including PDT or intravitreal anti-vascular endothelial growth factor (anti-VEGF) injection, and patients with any history of intraocular surgery within the past 6 months were excluded from the study. Because horizontal B-scan enhanced depth imaging (EDI) mode OCT images passing through the center of the fovea were used in CVI measurements, cases in which the PDT spot was extrafoveally placed without covering the fovea were excluded from the study in order to ensure standardization.

All the choroidal measurements were performed at the baseline visit and 3 months after hf-PDT. EDI-OCT images of a horizontal B-scan through the fovea were used for SFCT measurement. SFCT was manually measured using the measure distance tool of the device as the perpendicular distance between the outer portion of the hyperreflective line corresponding to the RPE to the hyporeflective margin corresponding to the chorioscleral junction. Then, the EDI-OCT images of a horizontal B-scan through the fovea were converted to binary images using ImageJ software (ImageJ program version 1.52 u bundled with 64-bit Java 1.80_112 (Wayne Rasband, National Institutes of Health, Bethesda, MD, USA, https://imagej.nih.gov/ij accessed on 4 April 2020). First, the total choroidal area was manually measured at a horizontally 3000 μm wide area with margins of 1500 μm away from both sides of the macula and from the RPE to the choroidoscleral border vertically. Then, the image was adjusted with the Niblack auto local thresholding method, in which white pixels represent the stromal area (SA) and dark pixels represent the luminal area (LA) [17]. The total choroidal area (CA), LA, and SA were measuredfor all groups. The CVI scores, which refer to the ratio of the LA to the CA, were also calculated. The main outcome measures in this study were the changes in the SFCT, total choroidal area, stromal area, luminal area, and CVI scores shortly after hf-PDT.

### 2.1. Photodynamic Therapy Protocol

The hf-PDT protocol was performed using a Visulas 690S PDT Laser System device (Zeiss/Humphrey 2003, Germany). Intravenous verteporfin was infused over 10 min at a dosage of 6 mg/m^2^. After 15 min from the initiation of the verteporfin infusion, a posterior pole laser lens (Volk^®^ area centralis, Volk Optical Inc, Mentor, OH, USA) was inserted into the affected eye, and a 689 nm laser was applied for 83 s. The applied light dose was 25 J/cm^2^ with an intensity of 300 mW/cm^2^. The spot size of the therapy was determined based on the choroidal hyperpermeability region on the ICGA. In the presence of PNV, the localization of the plaque and/or hot spot was also taken into consideration.

### 2.2. Statistical Analysis

The data were analyzed using SPSS version 15.0 software for Windows (SPSS Inc., Chicago, IL, USA). The Shapiro–Wilk test was used to test the normality of all data. Data are summarized as the mean ± standard deviation for continuous variables and frequencies (percentiles) for categorical variables. Based on the normality analyses, a paired-sample *t*-test or Wilcoxon test was applied to compare dependent variables, and an independent sample *t*-test or Mann–Whitney U test was used to compare the independent variables. To compare the level of change between the PNV and chronic CSCR groups after hf-PDT, the percentage of change was calculated for each parameter. Statistical significance was defined as *p* < 0.05.

## 3. Results

**Figure 1:**
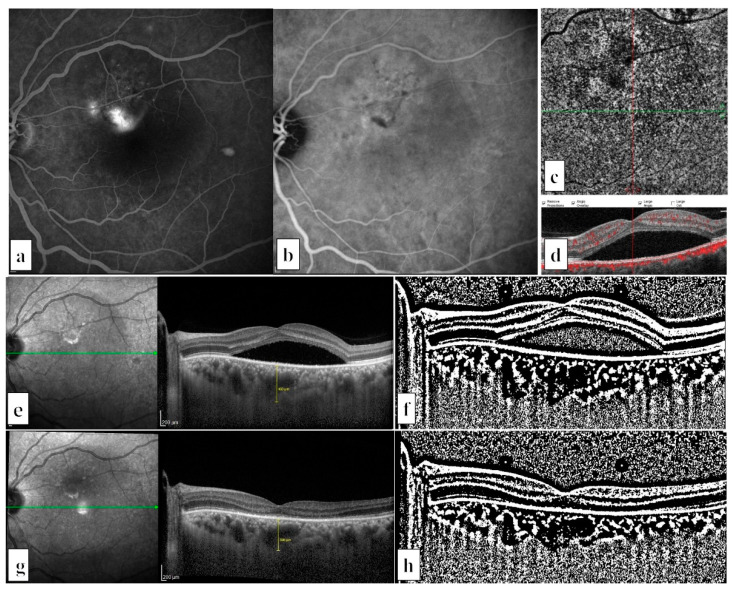
Multimodal fundus images of a patient with chronic central serous chorioretinopathy in his left eye: (**a**) Mid-late phase of fluorescein angiography showed two prominent fluorescein leakage areas on the superonasal macula with tiny window defects. (**b**) Mid-late phase of indocyanine green angiography revealed choroidal hyperpermeability in the same area. (**c**) Choriocapillaris slab of the optic coherence tomography angiography, and (**d**) B-scan flow overlay did not show any sign of choroidal neovascularization. (**e**) Enhanced depth imaging mode of optical coherence tomography (EDI-OCT) revealed subretinal fluid and increased subfoveal choroidal thickness (SFCT) (460 μm). (**f**) Binary EDI-OCT image before half-fluence photodynamic therapy (hf-PDT) (choroidal vascularity index = 76.0%, total choroidal area = 1.053, stromal area = 0.253, and luminal area = 0.800 mm^2^). (**g**) Three months after hf-PDT, EDI-OCT showed complete resorption of the subretinal fluid (SFCT= 390 μm). (**h**) Binary EDI-OCT image, three months after hf-PDT (choroidal vascularity index = 75.2%, total choroidal area = 0.789, stromal area = 0.196, and luminal area = 0.593 mm^2^).

**Figure 2:**
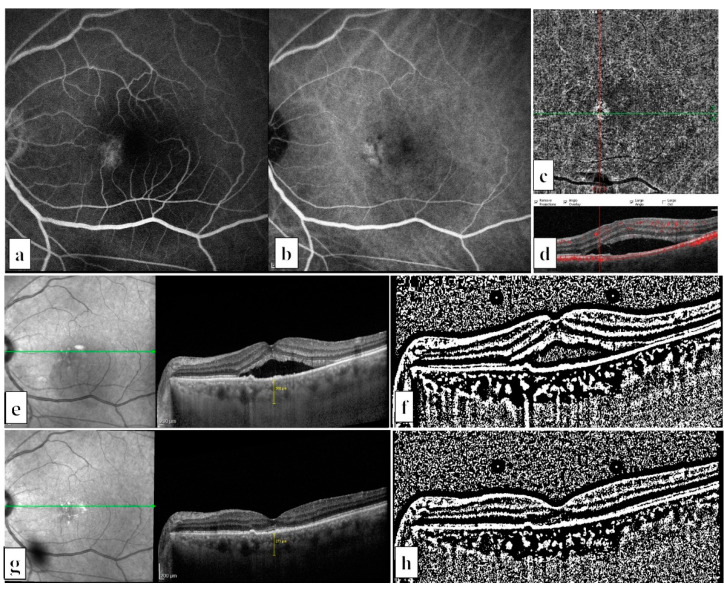
Multimodal fundus images of a patient with pachychoroid neovasculopathy in his left eye: (**a**) Mid-late phase of fluorescein angiography showed a leakage area located nasal to fovea. (**b**) Mid-late phase of indocyanine green angiography revealed a plaque appearance of type 1 choroidal neovascularization on the corresponding area. (**c**) Choriocapillaris slab of the optic coherence tomography angiography, and (**d**) B-scan flow overlay proved the presence of type 1 choroidal neovascularization. (**e**) Enhanced depth imaging mode of optical coherence tomography (EDI-OCT) revealed subretinal fluid, flat irregular pigment epithelium detachment (PED), and localized dilated Haller vessels under this PED. (**f**) Binary EDI-OCT image before half-fluence photodynamic therapy (hf-PDT) (choroidal vascularity index = 73.0%, total choroidal area = 0.826, stromal area = 0.223, and luminal area = 0.603 mm^2^). (**g**) Three months after hf-PDT, EDI-OCT showed complete resorption of the subretinal fluid. (**h**) Binary EDI-OCT image, three months after hf-PDT (choroidal vascularity index = 77.1%, total choroidal area = 0.812, stromal area = 0.186, and luminal area = 0.626 mm^2^).

We included 35 treatment naïve eyes of 35 chronic CSCR cases (4 women and 31 men) (Figure 1) and 18 treatment naïve eyes of 18 PNV cases (4 women and 14 men) (Figure 2). The participants’ mean ages were 47.1 ± 9.6 years in the chronic CSCR group and 55.0 ± 10.3 years in the PNV group (*p* = 0.007). The mean greatest linear diameter of the hf-PDT spot size was 3347 ± 1100 μm in the chronic CSCR group and 3250 ± 1037 µm in the PNV (*p* = 0.863) group.

Comparisons of the baseline and post hf-PDT characteristics between the PNV and chronic CSCR groups are given in Table 1. Regarding the baseline choroidal characteristics of the groups, the mean SFCT, CA, LA, and SA values were statistically significantly higher in the chronic CSCR group (*p* = 0.023, *p* = 0.004, *p* = 0.005, and *p* = 0.023, respectively). After hf-PDT, the differences between all choroidal parameters were no longer statistically significant (*p* > 0.05).

Comparisons of the baseline and post hf-PDT characteristics within the PNV and chronic CSCR groups are presented in Table 2. Three months after hf-PDT, the total CA reduced to 1.197 from 1.398 mm^2^ (*p* < 0.001), LA reduced to 0.896 from 1.041 mm^2^ (*p* < 0.001), and SA reduced to 0.301 from 0.357 mm^2^ (*p* < 0.001) in the CSCR group. Total CA reduced to 1.000 from 1.050 mm^2^ (*p* = 0.021), LA reduced to 0.737 from 0.770 mm^2^ (*p* = 0.031), and SA reduced to 0.263 from 0.280 mm^2^ (*p* = 0.073) in the PNV group. Changes in mean CVI values were not statistically significant in either the CSCR group (from 74.55% to 74.68%, *p* = 0.876) or the PNV group (from 73.17% to 73.65%, *p* = 0.416).

To compare the rate of changes between the PNV and chronic CSCR groups after hf-PDT, the percentage changes in all choroidal parameters were calculated (Table 3). The mean reductions in CA, LA, and SA were statistically higher in the chronic CSCR group (13.86%, 13.53%, and 14.11%, respectively) than in the PNV group (4.61%, 4.02%, and 5.74%; *p* = 0.001, *p* = 0.002, *p* = 0.031, respectively). The mean percentage change in SFCT and CVI values did not differ between groups (*p* = 0.083 and *p* = 0.677, respectively).

## 4. Discussion

In this study, all baseline choroidal parameters, except for the CVI, were lower in the PNV group. However, after one session of hf-PDT, all the differences in the choroidal measurement between groups disappeared. The reductions in the choroidal parameters (SFCT, CA, and LA) after hf-PDT were statistically significant in both groups. However, the percentage changes in these parameters were significantly different between the PNV and chronic CSCR groups. In the chronic CSCR group, hf-PDT induced an approximately 13–14% reduction in the SFCT and choroidal area measurements, whereas in the PNV group, these alterations were limited to approximately 4–8%. To the best of our knowledge, this study is the first to compare the direct impact of hf-PDT on choroidal vascular and stromal compartments in PNV and chronic CSCR patients. Our results revealed that although chronic CSCR and PNV are part of the same spectrum, their choroidal responses to hf-PDT vary. CSCR cases tended to show more pronounced responses and choroidal changes after hf-PDT.

The pachychoroid spectrum includes both uncomplicated pachychoroid cases, in which just a thick choroid is present without any pathological findings, and other types of diseases, in which pathological findings are seen in addition to pachychoroid features. PNV is characterized by reduced fundus tessellation, a thickened choroid, a dilated Haller’s layer vessel with the obliteration of the choriocapillaris underneath the CNV, choroidal vascular hyperpermeability, and RPE abnormality independent of CNV lesions [20,21]. PNV was first described by Pang and Freund in 2015 as a CNV in the presence of a thick choroid in patients with no previous history of CSCR attacks [3]. However, it may not always be possible to understand from the patient’s history or imaging findings whether the patient had a previous CSCR attack. Thus, previous studies evaluating this subgroup might have included CSCR cases complicated by CNV, especially in older patients. The exact stimuli triggering CNV formation underlying pachychoroid features remain unknown. According to the results of recent imaging studies using both dye angiography and OCTA, choroidal hyperpermeability areas, dilated vessels, and reduced perfusion of the choriocapillaris layer are colocalized. These findings indicated that an ischemic stimulus related to choriocapillaris attenuation and/or chronic mechanical disruption of the Bruch’s membrane might be associated with CNV development [2]. However, we still do not know which individuals are more prone to developing CNV over a diseased choroid or when it should be referred to as PNV if the case involves type 1 CNV over a dilated Haller layer or thick choroid [21]. Another question arising from the current literature is whether CSCR cases complicated by CNV are in the same spectrum as PNV.

Abnormal choroidal vasculature is the proposed causal factor in the pathogenesis of PSDs, and the CVI is a novel index directly measuring proportional changes in choroidal vascularity. Recent reports indicated that CVI can be used as a diagnostic marker and monitoring tool for choroidal diseases [22,23]. Furthermore, the CVI is a reliable research parameter due to its noninvasive nature, high repeatability, and the low influence of ocular and systemic parameters on it [18,19]. In this study, although other choroidal parameters showed remarkable reductions after hf-PDT, no changes in CVI scores were observed. This finding indicated that the stromal and luminal components decreased in a similar manner without causing a proportional change. The reduction in the stromal component is most likely due to the subsequent decrease in the stromal edema following a decrease in the hydrostatic pressure of choroidal vessels. Similarly, Iovino et al. observed no change in CVI at 1- and 3-month follow-ups after PDT in chronic CSCR cases [24].

Advances in imaging technology have led to a better understanding of the choroidal changes in CSCR and PNV. Lee et al. studied CVI in PSDs and reported that CSCR patients had significantly lower SAs than PPE, PNV, and myopic CNV patients and healthy controls [25]. They speculated that distinct pathogenic mechanisms may be involved in the development of PSDs. The primary insult that triggers PNV may be choriocapillaris ischemia related to advanced age and/or atherosclerosis and increased VEGF secretion, whereas CSCR may be caused by sympathetic activation and venous congestion [25]. Similarly, a recent study showed that higher SFCT and CVI values were obtained in CSCR cases than in PNV cases [26]. In this study, all baseline choroidal parameters, except for CVI, were lower in the PNV group. Even the fellow eyes of PNV and CSCR cases were shown to have an abnormal choroidal morphological structure [27].

These findings suggest that regardless of whether PNV and CSCR complicated by CNV are the same or different disease in the same spectrum, it seems that CNV and pachychoroid disease may be more localized choroidal pathologies rather than compromising the whole choroid in contrast to CSCR cases due to either vortex vein anastomosis or disease characteristics themselves [28]. However, this issue remains controversial. A recent study reported the remodeling of choroidal drainage routes through developed anastomoses at the watershed zones in PNV [28]. Thus, choroidal thickness reduces subsequent to established anastomoses, and CNV originates from these anastomotic vessels. This may be another possible explanation for the reason why PNV cases had lower choroidal measurement values. Because of these developed anastomoses, as the disease prolongs, pachychoroid features may become less obvious. Thus, CSCR cases might experience prominent favorable choroidal changes after PDT treatment because they have a relatively thick choroid and dilated vascular lumen, which are the main targets of PDT treatment, as previously reported [10]. However, regardless of the reason, the choroid may also benefit from hf-PDT in PNV, but not as much as in CSCR cases.

The results of studies speculating on the pathogenesis of these diseases are also supported by the reported clinical outcomes of PDT treatment for both pachy-CNV and CSCR cases. In CSCR, the complete resolution rate of the fluid is reported in 88–100% of cases [29,30,31], and the recurrence rate is reported as 2.9% [32], which are much better outcomes than those reported for pachy-CNV [12,33]. Although the results of previous studies have indicated good outcomes in pachy-CNV cases, some of these cases require anti-VEGF therapy in addition to PDT. A few studies have reported favorable results for PDT protocols in combination with anti-VEGF therapies in PNV [11,13]. However, only one study has evaluated the efficacy of full-fluence PDT monotherapy in PNV [12]. They reported complete fluid absorption in 85.7% of treated eyes after three months, but only 61% remained fluid-free over a 1-year follow-up period [12]. A recent study reported that three months after hf-PDT, the subretinal fluid completely regressed in 75.0% of the cases [33]. Although the results were promising, the rate of complete responses to PDT monotherapy did not reach the levels reached in CSCR studies, and the recurrence rate was relatively high. This situation may be helpful in explaining why combined therapy is required in a significant proportion of PNV cases. The choroidal hyperpermeability part of PNV may need to be treated with PDT, whereas CNV lesions may require intravitreal anti-VEGF injections. In a study evaluating choroidal anatomical alterations after PDT in CSCR cases, a small subgroup analysis of 13 eyes with CNV/PCV (11 CNV and 2 PCV) secondary to CSCR was performed [24]. Even though they reported reductions in all choroidal anatomical parameters in this subgroup, the decreases in the choroidal area and CVI measurements did not reach statistical significance due to the very limited number of CNV/PCV cases. In contrast to the aforementioned study, PNV eyes also showed significant reductions in CA and LA values after PDT in the present study. However, these decreases were not as prominent as those in CSCR subjects.

The major limitations of this study were the relatively small sample size, its retrospective design, and the short duration of the follow-up period. Therefore, the long-term results of the observed morphological alterations could not be evaluated. However, PNV cases may need combined anti-VEGF therapies during follow-up as opposed to CSCR cases, and the evaluation of the pure effect of hf-PDT on the choroid in the long term may not be possible in a large series of PNV patients because of combined anti-VEGF agents. Further prospective studies on larger cohorts and with longer follow-up periods are needed to validate our findings.

## 5. Conclusions

Although CSCR and PNV are in the same pachychoroid spectrum, their short-term choroidal structural response strength to hf-PDT varies. This finding may be due to the fact that PNV lesions have different structural properties that affect hf-PDT response behavior, in addition to its common pachychoroid features.

## Figures and Tables

**Table 1:** Comparisons of the baseline and post half-fluence photodynamic therapy characteristics between pachychoroid neovasculopathy (PNV) and chronic central serous chorioretinopathy (CSCR) groups.

	PNV *n* = 18		Chronic CSCR *n* = 35		*p* Value
	Mean	SD	Mean	SD	
Baseline choroidal characteristics					
Subfoveal choroidal thickness, µm	384.9	122.9	461.3	106.5	*0.023 ^a^*
Total choroidal area, mm^2^	1.050	0.341	1.398	0.429	*0.004 ^a^*
Luminal area, mm^2^	0.770	0.254	1.041	0.317	*0.005 ^b^*
Stromal area, mm^2^	0.280	0.090	0.357	0.121	*0.023 ^b^*
Choroidal vascularity index, %	73.17	2.29	74.55	2.64	0.067 ^a^
Post hf-PDT choroidal characteristics					
Subfoveal choroidal thickness, µm	353.1	116.9	399.6	109.9	0.160 ^a^
Total choroidal area, mm^2^	1.000	0.326	1.197	0.396	0.128 ^b^
Luminal area, mm^2^	0.737	0.239	0.896	0.304	0.128 ^b^
Stromal area, mm^2^	0.263	0.09	0.301	0.098	0.195 ^b^
Choroidal vascularity index, %	73.65	2.26	74.68	2.47	0.123 ^b^

Statistically significant *p* values (< 0.05) are italicized. *^a,b^* The test used: independent samples *t*-test or Mann–Whitney U test, respectively. hf-PDT: half-fluence photodynamic therapy.

**Table 2:** Comparisons of the baseline and post half-fluence photodynamic therapy (hf-PDT) characteristics within pachychoroid neovasculopathy and chronic central serous chorioretinopathy groups.

	Baseline		Post- hf-PDT		*p* Value
	Mean	SD	Mean	SD	
Pachychoroid neovasculopathy, *n* = 18					
Subfoveal choroidal thickness, µm	384.9	122.9	353.1	116.9	*0.002 ^a^*
Total choroidal area, mm^2^	1.050	0.341	1.000	0.326	*0.021 ^a^*
Luminal area, mm^2^	0.770	0.254	0.737	0.239	*0.031 ^b^*
Stromal area, mm^2^	0.280	0.090	0.263	0.09	0.073 *^a^*
Choroidal vascularity index, %	73.17	2.29	73.65	2.26	0.416 *^a^*
Chronic central serous chorioretinopathy, *n* = 35					
Subfoveal choroidal thickness, µm	461.3	106.5	399.6	109.9	*<0.001 ^a^*
Total choroidal area, mm^2^	1.398	0.429	1.197	0.396	*<0.001 ^b^*
Luminal area, mm^2^	1.041	0.317	0.896	0.304	*<0.001 ^b^*
Stromal area, mm^2^	0.357	0.121	0.301	0.098	*<0.001 ^b^*
Choroidal vascularity index, %	74.55	2.64	74.68	2.47	0.876 *^b^*

Statistically significant *p* values (<0.05) are italicized. *^a,b^* The test used: paired-sample *t*-test or Wilcoxon test, respectively.

**Table 3:** Comparisons of the percentage changes in choroidal parameters after half-fluence photodynamic therapy between pachychoroid neovasculopathy (PNV) and chronic central serous chorioretinopathy (CSCR) groups.

	PNV *n* = 18		Chronic CSCR *n* = 35		*p* Value
	Mean	SD	Mean	SD	
Percentage changes in the choroidal measurements					
Subfoveal choroidal thickness	8.04%	10.17	13.43%	10.66	0.083 ^a^
Total choroidal area	4.61%	7.73	13.86%	11.66	*0.001 ^b^*
Luminal area	4.02%	7.57	13.53%	12.56	*0.002 ^b^*
Stromal area	5.74%	13.01	14.11%	13.02	*0.031 ^a^*
Choroidal vascularity index	−0.70%	3.31	−0.26%	3.85	0.677 ^a^

Statistically significant *p* values (<0.05) are italicized. *^a,b^* The test used: independent samples *t*-test or Mann–Whitney U test, respectively.

## Data Availability

The datasets generated during and/or analyzed during the current study are available from the corresponding author on reasonable request.
